# Influence of inflammatory response on infarct size and microvascular obstruction estimated by cardiac magnetic resonance in patients with ST-elevation myocardial infarction

**DOI:** 10.1186/1532-429X-17-S1-P160

**Published:** 2015-02-03

**Authors:** Justyna Rajewska-Tabor, Magorzata Pyda, Anna Kociemba, Magdalena Janus, Magdalena Lanocha, Andrzej Siniawski

**Affiliations:** I Cardiology Clinic, University of Medical Sciences in Poznan, Lusowo, Poland; Magnetic Resonance Department, University of Medical Sciences in Poznan, Poznan, Poland

## Background

The inflammatory response during ST-segment elevation myocardial infarction (STEMI) has been shown to influence the clinical outcome. Moreover, infarct size (IS) and microvascular obstruction (MVO) predict major adverse events in patients with STEMI.

The aim of the study was to compare the inflammatory response measured by C-reactive protein (CRP) serum concentration and the number of white blood cells (WBC) with the infarct size and MVO estimated by CMR.

## Methods

We examined 85 patients (mean age 59±11 years; 59 males and 26 females) with acute STEMI. CRP and white blood cells were measured at the admission to the hospital. CMR examinations were performed on a 1.5 T scanner (Siemens, Avanto) using an eight-channel phased-array coil combined with 4-6 elements of spinal coil within 3 days after STEMI. Cine imaging with steady-state free precession and late gadolinium enhancement (LGE) were performed in the long axis and the contiguous short axis slices to evaluate myocardial function, IS and MVO. Infarct size was defined as an area greater than 50% of the maximal signal intensity within LGE (FWMH - full-width half maximum). MVO was diagnosed as an area of contrast hypoenhancement within the infarct zone and was included in the assessment of IS. IS and MVO were determined by planimetry and a summation of discs method.

## Results

The ejection fraction in the examined population was 54.5±10%, infarct size: 24.57±20.79g and MVO: 2.02±4.7g. WBC and CRP levels were measured at the admission to the hospital (WBC: 11.34±3.34 mld/l and CRP serum level: 21.58±26.2 mg/dl). There were 27 patients with small (≤10% of myocardium) infarct size and 58 patients with bigger IS (>10%). Despite a significant difference in ejection fraction in both groups (60.33±7.79% and 51.75±9.98%; p=0.002), no significant differences in the inflammatory response were noted in either of the groups. However, we observed a weak correlation between CRP serum level and infarct size (p=0.03; r=0.23) and a stronger one between the leukocyte number and the infarct size (p=0.000004; r=0.72). The number of WBC and the CRP serum level also correlated significantly with the size of MVO (p=0.014; r=0.45 and p=0.0004; r=0.69).

## Conclusions

Inflammatory response during STEMI influences infarct size and microvascular obstruction measured by CMR.

## Funding

N/A.

